# β2-glycoprotein I promotes the clearance of circulating mitochondria

**DOI:** 10.1371/journal.pone.0293304

**Published:** 2024-01-25

**Authors:** Swapan Kumar Dasgupta, Jahnavi Gollamudi, Stefanie Rivera, Ross A. Poche, Rolando E. Rumbaut, Perumal Thiagarajan

**Affiliations:** 1 Center for Translational Research on Inflammatory Diseases (CTRID), Michael E. DeBakey Veterans Affairs Medical Center and Departments of Pathology, Baylor College of Medicine, Houston, Texas, United States of America; 2 Department of Medicine, Baylor College of Medicine, Houston, Texas, United States of America; 3 Department of Medicine Integrative Physiology, Baylor College of Medicine, Houston, Texas, United States of America; Khalifa University of Science and Technology, UNITED ARAB EMIRATES

## Abstract

β2-glycoprotein I (β2-Gp1) is a cardiolipin-binding plasma glycoprotein. It is evolutionarily conserved from invertebrates, and cardiolipin-bound β2-Gp1 is a major target of antiphospholipid antibodies seen in autoimmune disorders. Cardiolipin is almost exclusively present in mitochondria, and mitochondria are present in circulating blood. We show that β2-Gp1 binds to cell-free mitochondria (CFM) in the circulation and promotes its phagocytosis by macrophages at physiological plasma concentrations. Exogenous CFM had a short circulation time of less than 10 minutes in mice. Following infusion of CFM, β2-Gp1-deficient mice had significantly higher levels of transfused mitochondria at 5 minutes (9.9 ± 6.4 pg/ml versus 4.0 ± 2.3 pg/ml in wildtype, p = 0.01) and at 10 minutes (3.0 ± 3.6 pg/ml versus 1.0 ± 0.06 pg/ml in wild-type, p = 0.033, n = 10). In addition, the splenic macrophages had less phagocytosed CFM in β2-Gp1-deficient mice (24.4 ± 2.72% versus 35.6 ± 3.5 in wild-type, p = 0.001, n = 5). A patient with abnormal β2-Gp1, unable to bind cardiolipin, has increased CFM in blood (5.09 pg/ml versus 1.26 ± 1.35 in normal) and his plasma induced less phagocytosis of CFM by macrophages (47.3 ± 1.6% versus 54.3 ± 1.3, p = 0.01) compared to normal plasma. These results show the evolutionarily conserved β2-Gp1 is one of the mediators of the clearance of CFM in circulation.

## Introduction

β2-glycoprotein I (β2-Gp1), a cardiolipin and other anionic phospholipid-binding glycoprotein, is present at a concentration of 50–200 μg/ml in normal plasma [1]. Its precise physiological function is not known. It has been conserved in evolution across species from insects to humans. It is composed of five complement control protein (CCP) domains each containing approximately 60 amino acids with a relatively invariant arrangement of 2 disulfide bonds [2]. CCP5 diverges from the norm for CCPs, in that it has a unique pattern of an additional disulfide bridge and has an evolutionarily conserved positively charged motif CKNKEKKC, which mediates cardiolipin binding. In crystal structure, it has a “stretched” form with the first four CCP domains arranged like beads in a string, with domain 5 at a right angle to the other domains, resembling a “fishhook” or a “hockey stick”. In solution, β2-Gp1 exist in multiple conformations including a “closed” or an “open” form [3–5].

Cardiolipin is present almost exclusively in the mitochondrial membrane and is essential for the structural integrity and optimal functioning of the respiratory chain components [6]. During mitophagy, cardiolipin undergoes transbilayer movement from inner to outer bilayer [7]. In the outer layer, it provides binding platform for the specific recruitment of signaling molecules involved in mitophagy [8]. Mitochondria can be shed from cells through various mechanisms [9–11]. Recent investigations show that mitochondria are found in extracellular space in normal physiological condition. The circulating blood contains mitochondria as intact organelles or in enclosed vesicles [12–16]. In the extracellular space, mitochondria can have harmful proinflammatory effects and a clearance mechanism must exist for normal homeostasis. Since β2-Gp1 binds to cardiolipin, we investigated its potential role in the clearance of cardiolipin-expressing CFM. We show that the evolutionarily conserved β2-Gp1 promotes the clearance of cardiolipin-expressing extracellular mitochondria from the circulation by macrophages.

## Materials and methods

### Reagents

Monoclonal Alexa Fluor 647-labelled Anti-translocase of outer membrane (TOMM20) antibody, rabbit polyclonal antibodies to human albumin and cytochrome oxidase subunit 4I1 (COXIV) were obtained from Abcam. Fluorescein isothiocyanate (FITC)-labeled and phycoerythrin (PE)–labeled monoclonal antiCD11b antibodies were from Beckman-Coulter Life Sciences. Cardiolipin (bovine heart) was purchased from Avanti Polar Lipids Inc. Lysotracker Green DND-26, Alexa Fluor-phalloidin, DAPI (4’,6-Diamidino-2-Phenylindole, Dihydrochloride), G418, rotenone, 10-N-nonyl acridine orange (NAO), primers for PCR, secondary peroxidase labelled antibodies and all other standard chemicals were obtained from ThermoFisher Scientific. β2-Gp1 was isolated from normal plasma and labelled with Alexa fluor 488 hydrazide as described previously [17]. Plasmid m-Cherry mito-7 which has Cytochrome C Oxidase Subunit 8A fused to fluorescent protein mCherry (# 55102) was obtained from Addgene. Bacterial endotoxin (Lot 040M4105) was obtained from Sigma-Aldrich and purified recombinant LC3B was obtained from Abcam.

### Cells

THP-1 cells and human embryonic kidney (HEK293) cells were obtained from ATCC. THP-1 cells were grown in tissue culture medium RPMI 1640 containing 10% fetal bovine serum. The cells were treated with phorbol 12-myristate 13-acetate (150 ng per ml) for 48 h to differentiate towards monocytes. HEK293 cells were transfected with plasmid m-Cherrymito 7 using X-trmeGENE HP DNA transfection regent (Roche Diagnostics) and transfected cells were selected with G418 (400 μg/ml). Thioglycollate elicited peritoneal macrophages were isolated as described before [18].

### Mice

C57BL/6J mice were purchased from the Jackson Laboratory (Bar Harbor, ME). Embryonic stem (ES) cells with knockout of β2-Gp1 were obtained from KOMP (knockout mouse project). The targeting vector contains a ZEN-Ub**1** cassette consisting of a lacZ-p(A) reporter and hUbCpro-neo-p(A) selectable marker flanked by loxP sites, which following homologous recombination replaces the exons 1-7with construct depicted in (S1A Fig in S1 File). We generated β2-Gp1-deficient mice in Baylor College of Medicine animal core facilities. The wild type primers recognize sequences situated between exon **1** and **2** (red arrows) and amplify a 400 bp DNA fragment whilst the KO primers recognize sequences situated between Neo cassette and exon 7 and amplify a 605 bp DNA fragment (S1B Fig in S1 File). No immunoreactive β2-Gp1 was detected in the β2-Gp1-deficient mice (S1C Fig in S1 File). The primer sequences are described in S1 Table in S1 File. Mito::mKate2 mice, which express the bright red fluorescent mitochondria have been described previously [19].

### β2-Gp1 binding to mitochondria

Mitochondria were isolated from HEK293 cells [20]. Mitochondrial suspensions (1–50 μg) were added to wells of 96 well microtiter plates (Nunc MaxiSorb flat bottom) and incubated overnight at 4°C. The wells were washed gently, blocked in 5% non-fat milk in Tris-buffered saline, pH 7.5, and incubated with various concentrations of β2-Gp1. Bound β2-Gp1 was quantified by a laboratory made polyclonal rabbit antibody to β2-Gp1 and peroxidase labelled goat antirabbit antibody.

The β2-Gp1 binding to mitochondria was also assessed in a flow cytometer (Cytoflex LX Coulter; Beckman-Coulter Life Sciences) using Alexa-488-labelled β2-Gp1. The acquisition and data analysis was performed using CytExpert Software after calibration with standards (Spherotech fluorescent particles) for accurate identification of subcellular-sized particles as described previously in detail [21]. The mitochondrial gate was set by size and by positive fluorescence using Alexa 647-labelled monoclonal antibody to mitochondria-specific protein TOMM20 [21]. Various concentrations of Alexa488-labelled β2-Gp1 were incubated with isolated mitochondria in suspension in phosphate-buffered saline for 30 minutes at room temperature and washed three times before analysis. To resolve mitochondria from background, only the populations that were positive for TOMM20 were gated and analyzed for β2-Gp1 binding (green fluorescence). Fluorescence and light-scattering intensity are expressed as arbitrary units on a logarithmic scale. Overall, 10,000 events per sample were acquired to ensure adequate mean fluorescence levels. Some preparations of mitochondria were incubated with rotenone (0.1 μM) for 30 minutes. In some experiments mitochondria are incubated with LC3B (25 μg/ml). Unlabeled β2-Gp1 (25 μg/ml), annexin A5 (25 μg/ml) or Rotenone (μM) before the addition of Alexa-488-labelled β2-Gp1.

Mitochondria were also isolated from peripheral blood as described before [12]. An aliquot (20 ug/per lane) was subjected SDS-PAGE, transferred to PVDF membrane and immunoblotted with antibodies to β2-Gp1, albumin, and mitochondria specific protein COXIV.

### β2-Gp1-dependent phagocytosis of mitochondria by macrophages

For assessing phagocytosis by fluorescence microscopy, mouse peritoneal macrophages were isolated and plated onto 35 mm dish with 10 mm diameter glass bottom microwell for 48 hours. The adherent macrophages washed and incubated with red fluorescent mitochondria (50 μg/per well) isolated from the liver of mito::mKate2 mouse at 37°C for 1 h in the presence or absence of 50 μg/ml of β2-Gp1 in serum-free medium. The macrophages were washed quickly with 0.05% trypsin-EDTA to promote detachment of surface bound mitochondria and then were washed twice in serum-free medium. The cells were fixed in 4% formaldehyde (10 minutes), washed and permeabilized with 0.1% Triton X 100 (5 minutes) and stained with Alexa-488-phalloidin. In some experiments, the nuclei are stained with DAPI, and the lysosomes were stained with lysotracker green DND26. The cells were examined in fluorescent microscope (Olympus Fluoview FV3000) and the images were analyzed by FV31S-SW Viewer software (Olympus Life sciences).

For assessing phagocytosis by flowcytometry, human macrophages, derived from THP-1 cells (stimulated with phorbol 12-myristate 13-acetate (150 ng/ml) for 3 days, were plated on 12 well tissue culture plate (1 × 10^6^/well). Red fluorescent human mitochondria, isolated from HEK293 cells transfected with the plasmid m-Cherrymito 7, were incubated with macrophages at 37°C for 30 minutes in the presence or absence of 50 μg/ml of β2-Gp1 as described for the peritoneal macrophages. Cells were treated with 0.05% trypsin-EDTA to promote detachment surface bound mitochondria. The trypsin was neutralized with fetal calf serum and the cells were washed twice in serum-free medium and incubated with FITC-labeled anti-CD11b monoclonal antibody (5 μg/ml). The THP-1 cells were gated by their scattering and by CD11b reactivity (FITC fluorescence). The CD11b-positive cells were analyzed for red fluorescence (mCherry) and the percentage of red fluorescent macrophages to total CD11b-positive macrophages was determined. Phagocytosis in the absence of β2-Gp1, which varied from 10–25% in different experiments, was defined as 1 for comparison.

### Quantification of mitochondria in blood

Circulating CFM were isolated from mouse and human plasma as described before [12]. Mitochondrial DNA (mtDNA) was isolated using a QIAamp DNA Blood Mini Kit (Qiagen) and amplified by quantitative polymerase chain reaction (qPCR) with primers (S1 Table in S1 File), using SsoAdvanced Universal SYBR Green Supermix (Bio-Rad). These primers and probes are specific for detecting mouse or human mtDNA [22]. Sequencing the mtDNA of inbred strains of mice has shown the presence of single nucleotide polymorphisms (SNP) that are unique among the strains. The mtDNA of BALB/c differs from the C57B at position 9461 in the ND3 gene (C9461T) and at position 9348 in the COIII of mtDNA (A9348G) [23]. We designed allele specific primers for each strains of mice with the mismatch at the 3’ ends as described previously [24] (S1 Table in S1 File). The qPCR reaction contained 10 μl 2× SsoAdvanced Universal SYBR Green Supermix, 2 μl primers (10 μM) and 8 μl of DNA (0.5–5 ng μl−1) for a final volume of 20 μl. A standard curve was generated with known quantities of mtDNA and the corresponding Ct values obtained by quantitative polymerase chain reaction. The accuracy of the assay was demonstrated by measuring serially diluted mtDNA preparations from 1 pg/ml to 10 ng/ml (S2 Fig in S1 File).

### Estimation of survival time of mitochondria in circulation

To measure reproducibly the circulation time in vivo, we modified previously described intravital thrombosis models [25]. In anesthetized mice, a midline incision is made in the neck, and the trachea is exposed by blunt dissection. A tracheotomy tube is passed through a small incision in the trachea and secured in place with a silk suture. A carotid artery is exposed and clamped with a vascular clamp and a catheter containing heparinized saline is advanced through a small incision in the artery. Thereafter, an internal jugular vein is exposed and a catheter containing heparinized saline is advanced through a small incision in the vein and secured with a suture. The skin incision is closed, and the animal is kept in surgical plane anesthesia. Mitochondrial suspensions, isolated from HEK293 cells (for human) or liver of BALB/c mice, were infused into β2-glycoprotein I-deficient mice or wildtype control mice via the jugular vein catheter. Blood (200 μl) drawn from the carotid artery catheter at 1, 5 and 10 minutes. The blood is centrifuged at 1000 g for 15 minutes to remove cells and the cell-free supernatant further centrifuged at 12000 g for 10 minutes to sediment the mitochondria. The mitochondrial DNA was extracted and quantified by qPCR as described above.

### Phagocytosis of mitochondria by mouse macrophages in vivo

Mitochondria, isolated from the liver of mito::mKate2 mice were infused intravenously to recipient β2-Gp1-deficient mice and wildtype controls. After 30 minutes the mice were euthanized, and the spleen was harvested, and single cell suspensions were prepared. Phagocytosed mitochondria within the macrophages were quantified by flow cytometry as described earlier.

### Clinical summary

We previously described a patient who is compound heterozygous for two mutations in the phospholipid domain. (p.Cys316Gly in exon 7 and p.Trp326Ser in exon 8) [26]. The mutant β2-Gp1 was present in normal quantities in his plasma but did not bind to cardiolipin. He had recurrent deep vein thrombosis and pulmonary embolism at age 28 and a thrombotic stroke at age 35, with no other identifiable risk factor for a hypercoagulable state. We obtained plasma from fresh blood in 2002, measured the CFM and tested the effect of his plasma mitochondrial uptake. Mitochondria (500 μg/ml), were isolated from HEK293 cells, labeled with mitoTracker green (200 nM) and incubated with washed human peripheral blood-derived monocytes (10^5^/ml) for one hour at 37°s normal or patient plasma (50% v/v). The cells were washed, and the intracellular phagocytosed mitochondria were determined by flow cytometry as described earlier.

### Statistical methods

Comparisons between individual groups were performed with T-test using Microsoft Excel. All experiments will be biological triplicate or more. A probability value (p) of 0.05 or less was considered statistically significant.

### Study approval

The Institutional Animal Care and Use Committee of Baylor College of Medicine approved all animal protocols and the human blood was obtained after an informed written consent under a protocol approved by the Institutional Review Board of Baylor College of Medicine.

## Results

### β2-Gp1 interacts with cardiolipin-expressing mitochondria

**Fig 1:**
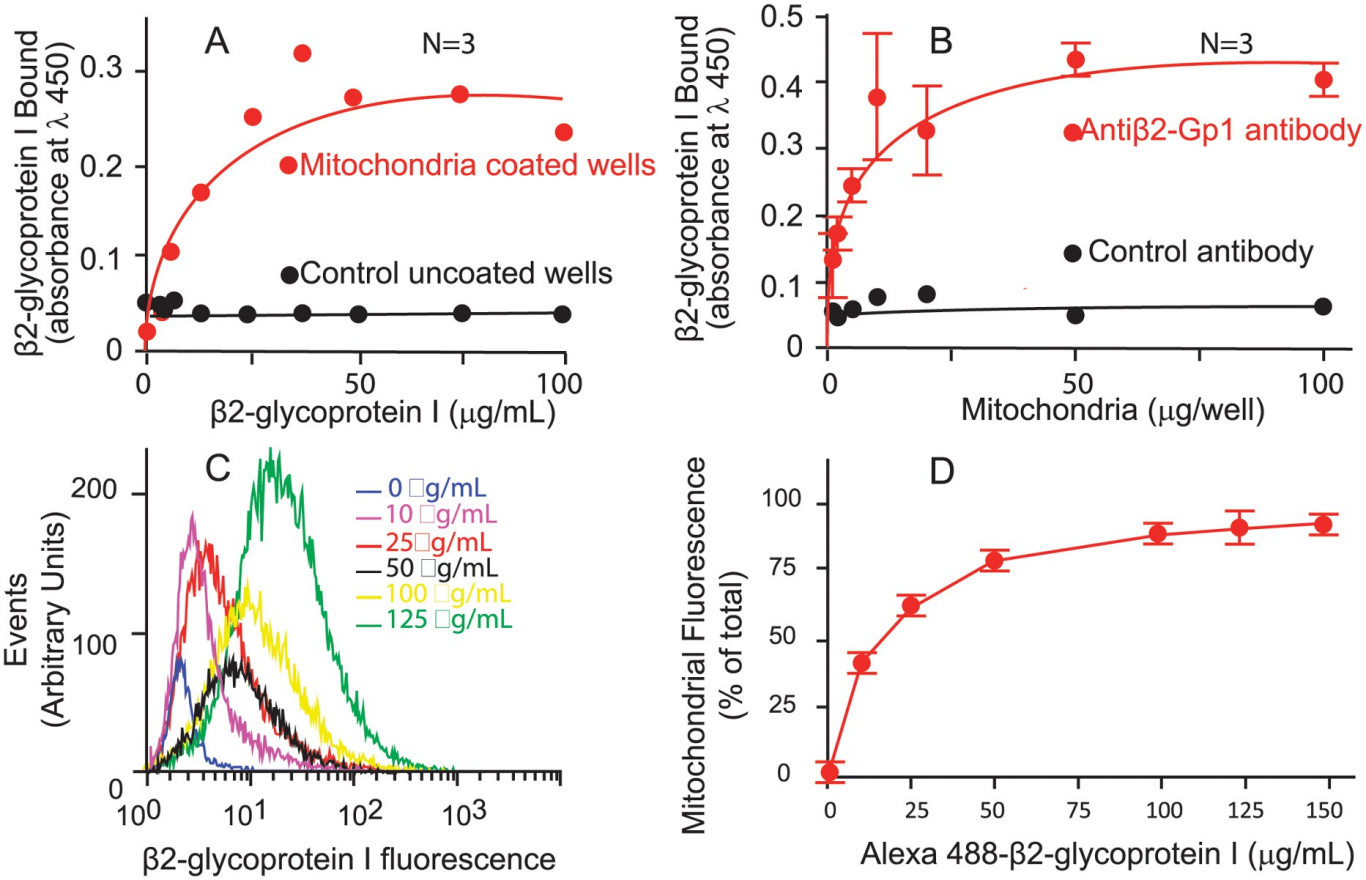
β2-Gp1 binding to mitochondria. Panel A, wells of microtiter plates were coated with mitochondria (100 μg per well) and various concentrations of β2-Gp1 were added. Bound β2-Gp1 was quantified by an antibody to β2-glycoprotein I by ELISA. Panel B, Various amounts of mitochondria were immobilized, blocked and incubated with β2-Gp1 (50 μg/ml) and the binding was quantified by ELISA. Panels C and D, mitochondria were incubated with various concentrations of Alexa Fluor 488-labelled β2-Gp1 and Alexa 647-labeled monoclonal antibody to TOMM20 in suspension and the binding was analyzed by flow cytometry.

As shown in Fig 1A, β2-Gp1 bound to immobilized mitochondria in a concentration-dependent manner and reaching saturation at 50–100 μg/ml in ELISA. Binding was dependent on the number of mitochondria added to the plate (Fig 1A and 1B). We also measured the binding of Alexa 488-labeled β2-Gp1 by a flow cytometric method. As shown in Fig 1C and 1D, there was a concentration-dependent binding of β2-Gp1 to mitochondria. The mean fluorescence intensity and percentage of positive mitochondria were increased with increasing concentration of β2-Gp1.

**Fig 2:**
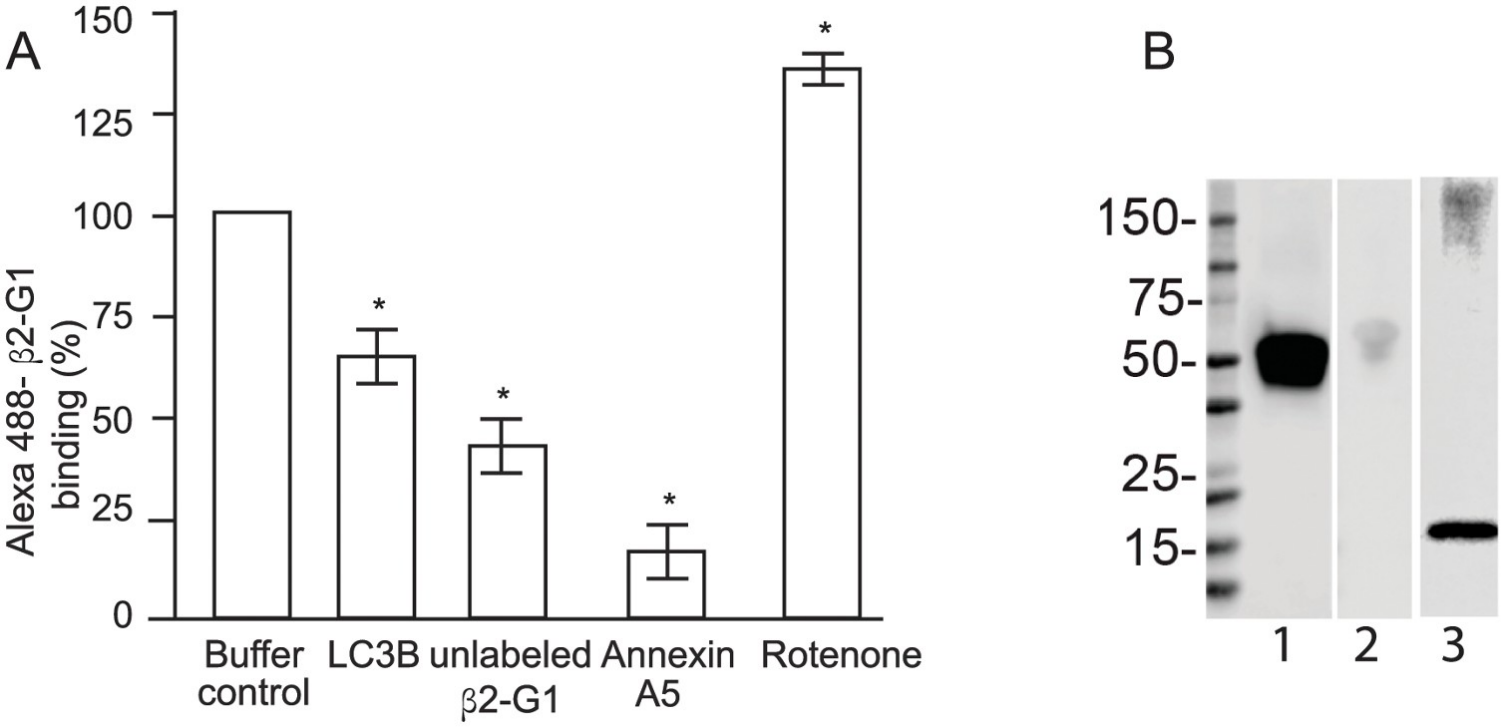
β2-Gp1 binding to mitochondria. In Panel A, binding of Alexa 488-β2-Gp1 to isolated mitochondria in the presence of buffer, LC3 (25 μg/ml), unlabeled β2-Gp1 (25 μg/ml), annexin A5 (25 μg/ml) or rotenone (1 μM) as determined by flow cytometry. * p<0.01., Panel B. β2-Gp1 in circulating mitochondria. Mitochondria were isolated from blood, washed, subjected SDS-PAGE (20 μg/lane), transferred to PVDF membrane and immunoblotted with antibodies to β2-Gp1 (lane 1), albumin (lane 2) and mitochondria specific protein COX4 subunit (lane 3).

Cardiolipin is the major anionic phospholipid (>90%) in mitochondria [27] and the binding of β2-Gp1 to mitochondria was inhibited by the cardiolipin binding protein LC3B [28] and by the anionic phospholipid binding protein annexin A5 [29] (Fig 2A). Furthermore, rotenone, an inhibitor of complex I of the mitochondrial respiratory chain, induced externalization of cardiolipin [7] and β2-Gp1 binding to mitochondria increased following rotenone treatment (Fig 2A). These results are consistent with β2-Gp1 binding to cardiolipin in mitochondria.

To assess whether β2-Gp1 binds to mitochondria in circulating blood, we isolated mitochondria from normal blood [30], washed thoroughly and immunoblotted with antibodies to β2-Gp1, albumin and mitochondrial marker COXIV. A significant amount of mitochondria-bound β2-Gp1 was detected compared to trace amounts of albumin (Fig 2B). This is significant as the concentration of albumin (~40 mg/ml) is 200-800-fold greater than the plasma concentration of β2-Gp1 (50–200 μg/ml).

### β2-Gp1 promotes phagocytosis of mitochondria by mouse macrophages

**Fig 3:**
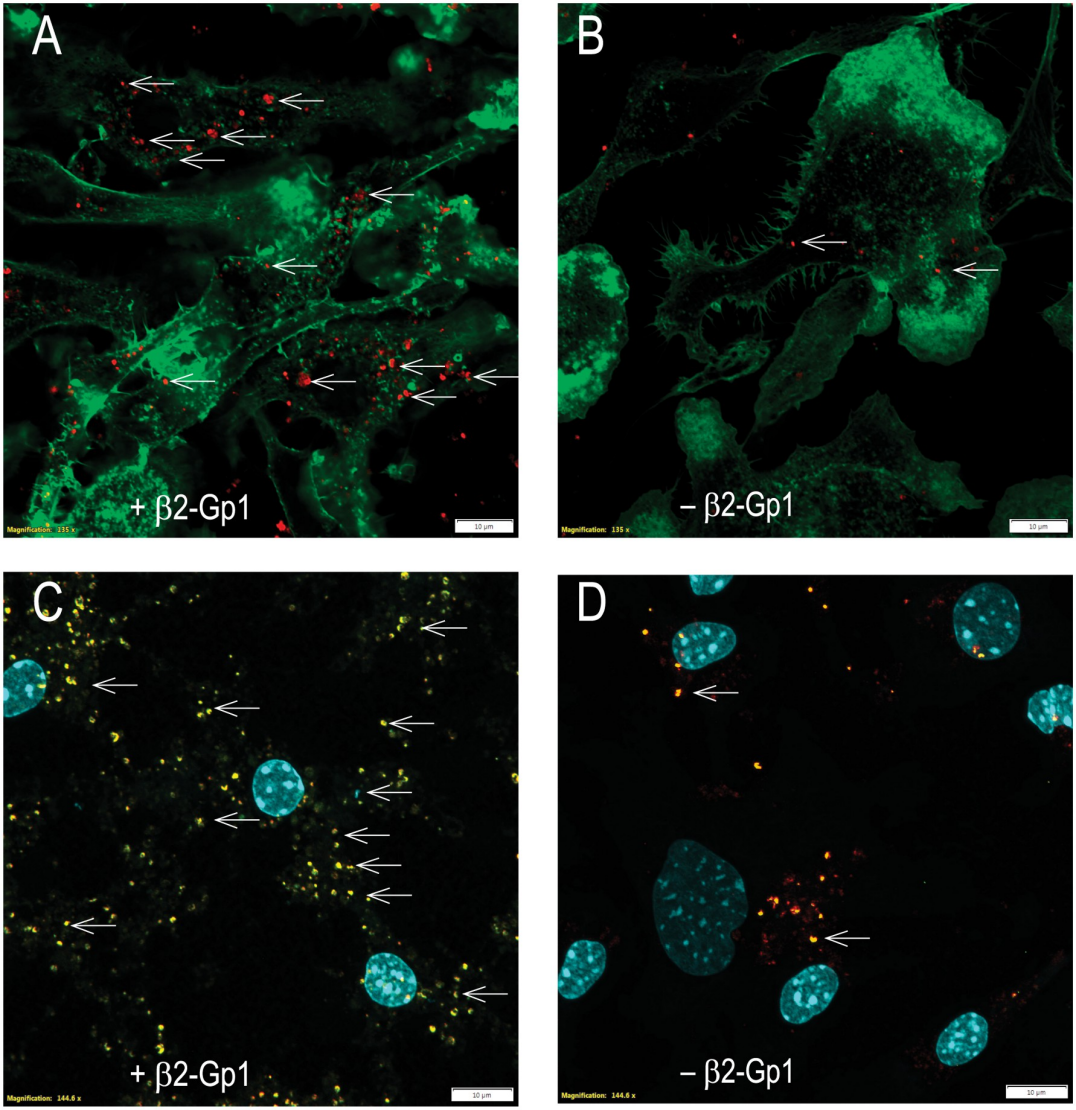
Phagocytosis of mitochondria by mouse macrophages. Panels A and B. Fluorescent mitochondria from the liver of mito::mKate2 mice were isolated and incubated with mouse peritoneal macrophages with (Panel A) and without (Panel B) β2-Gp1. The macrophages were stained with Alexa fluor 488-Phalliodin (for cytoplasm). The arrows point to red fluorescent mitochondria within the cells. Panels C and D. Fluorescent red mitochondria from mito::mKate2 mice were incubated with peritoneal macrophages with (Panel C) and without (Panels D) β2-Gp1. The peritoneal macrophages were stained with DAPI (for nucleus) and Lysotracker Green DND-26 (for lysosome). The arrows points to lysosomes (green) and lysosomes colocalized with engulfed mitochondria (yellow). Representative figures of at least 4 or more independent biological replicates.

We evaluated mouse peritoneal macrophages in the phagocytosis of exogenous mitochondria. When examined under the microscope, addition of β2-Gp1 showed significant increase in the uptake fluorescent mitochondria by the macrophages (Fig 3A and 3B). β2-Gp1-dependent mitochondrial uptake reached a maximum within 30 minutes. On further incubation, engulfed mitochondria and β2-Gp1 were localized to the lysosome (Fig 3C and 3D), as shown by its colocalization with the lysosomal marker lysoTracker green. These results show β2-Gp1 promotes the uptake and for delivery to the lysosome.

### β2-Gp1 promotes phagocytosis of mitochondria by human THP-1-derived macrophages

**Fig 4:**
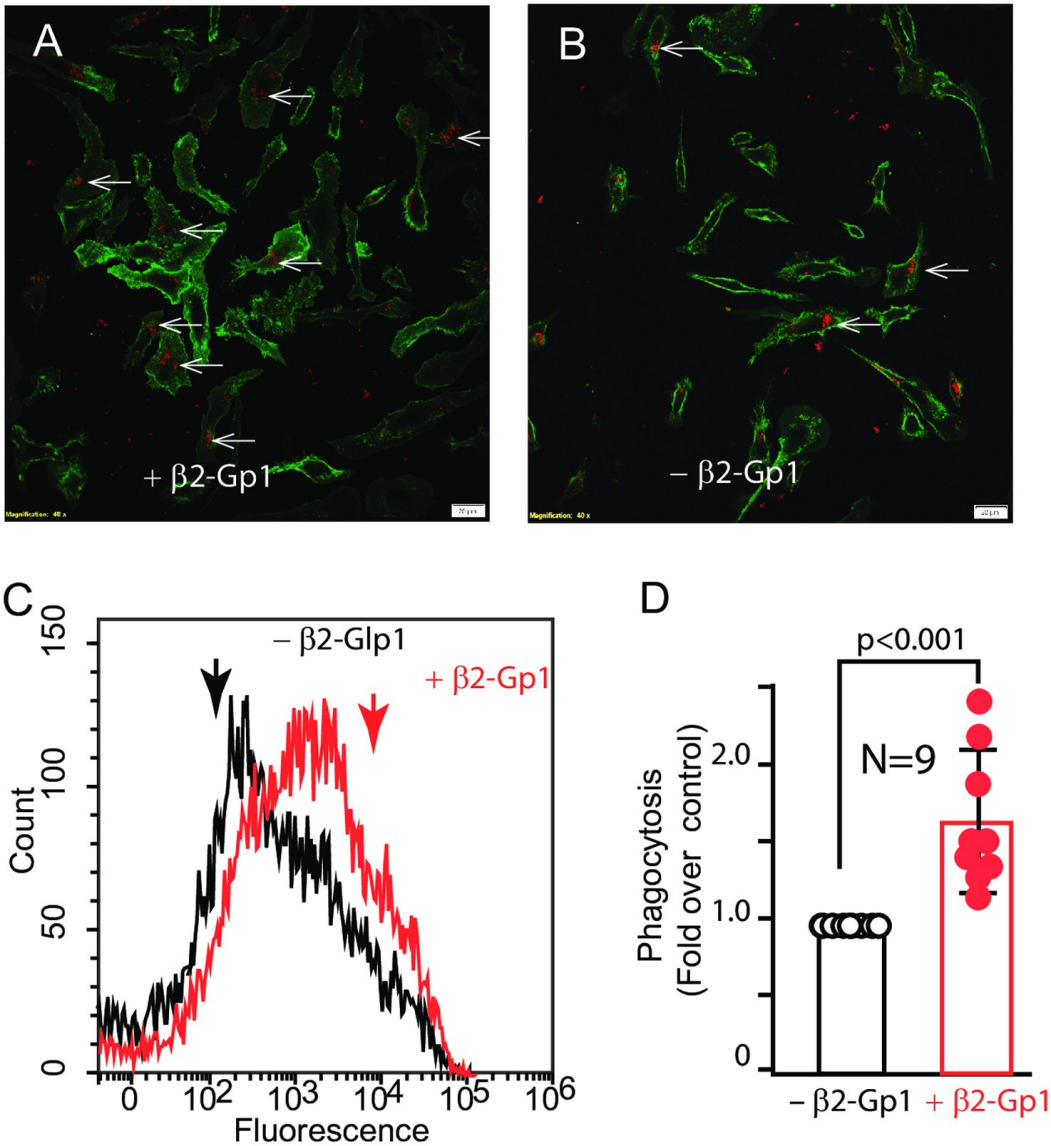
Phagocytosis of mitochondria by mouse macrophages. Panels A and B, Fluorescent human mitochondria (labelled with Tomm20) were incubated with THP-1-derived macrophages in presence (Panel A) or absence (Panel B) of β2-Gp1 for 30 minutes and analyzed by fluorescence microcopy. The arrows point to intracellular mitochondria. Representative figures of at least 4 or more independent biological replicates. Panels C, flowcytometric analysis of internalized human mitochondria. Panel D, plot of the effect of β2-Gp1 on the phagocytosis of mitochondria. The white bar are 10 microns in length (P = <0.001, n = 9).

We confirmed β2-Gp1-mediated mitochondrial phagocytosis in human macrophages derived from THP-1 cells by flowcytometric analysis. Phagocytosis in the absence of β2-Gp1, which varied from 10–25% in different experiments and it was arbitrarily defined as 1. Addition of β2-Gp1 at physiological concentration (50 μg/ml) promoted phagocytosis of mitochondria by human macrophages (Fig 4, Panels A-D).

### Increased CFM and impaired clearance of CFM in β2-Gp1

**Fig 5:**
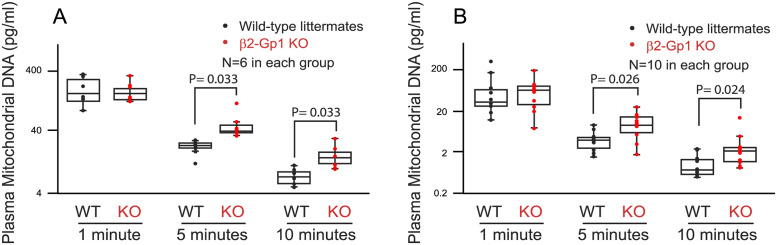
Clearance of CFM in mice. Mitochondria were from HEK293 cell line (Panel A), or liver of BALB/c mice (Pane B) were infused via the jugular vein to β2-Gp1-deficient mice or wildtype controls in C57 background at time 0. Blood samples were collected from the carotid artery at indicated times and the circulating plasma mitochondria were quantified by measuring DNA content by qPCR with human or BALB/c mitochondrial DNA-specific primers.

We isolated circulating CFM from murine plasma and quantified their levels by measuring mitochondrial DNA content by qPCR using unique sequences in mitochondrial genome not present in the nuclear genome [31]. β2-Gp1-deficient mice had increased level of circulating mitochondria in the blood suggesting a defect in clearance (69 ± 34 pg/ml versus 20.0 ± 8.6 pg/ml in wildtype, P = 0.0012). Six hours after endotoxin infusion, there was further increase in CFM in β2-Gp1-deficient mice compared to wildtype control mice (206 ± 51 pg/ml versus 75.0 ± 41 pg/ml in wildtype, P = 0.001. When exogenous CFM were infused to mice were cleared rapidly within 15 minutes. To reproducibly measure the circulation time of infused mitochondria in vivo, we employed an intravital preparation to measure the rapid disappearance of infused mitochondria [25]. Exogenous CFM were infused to anesthetized via the jugular vein. At 5 minutes following infusion, the β2-Gp1-deficient mice had higher levels of human mitochondria compared to the wildtype controls (54.5 ± 31.7 pg versus 22.2 ± 5.9 pg, p = 0.033, n = 7). At 10 minutes, infused exogenous mitochondria were still detected at higher levels (15.38 ± 8.2 pg) in β2-Gp1-deficient mice while in wildtype control the levels were down to the limits of detection (7.13 ± 2.2 pg p = 0.033, n = 7). (Fig 5, Panel A). We also developed an allele specific qPCR to measure the circulation of mitochondria from BALB/c strain of mice in B56 background. As shown in Fig 5, Panel B, following infusion of mitochondria from BALB/c mice, β2-Gp1 KO mice in B6 background had significantly higher levels of mitochondria at 5 minutes (9.9 ± 6.4 pg/ml versus 4.0 ± 2.3 pg/ml in wildtype, p = 0.01) and at 10 minutes (3.0 ± 3.6 pg/ml versus 1.0 ± 0.06 pg/ml in wild-type, p = 0.033, n = 10).

**Fig 6:**
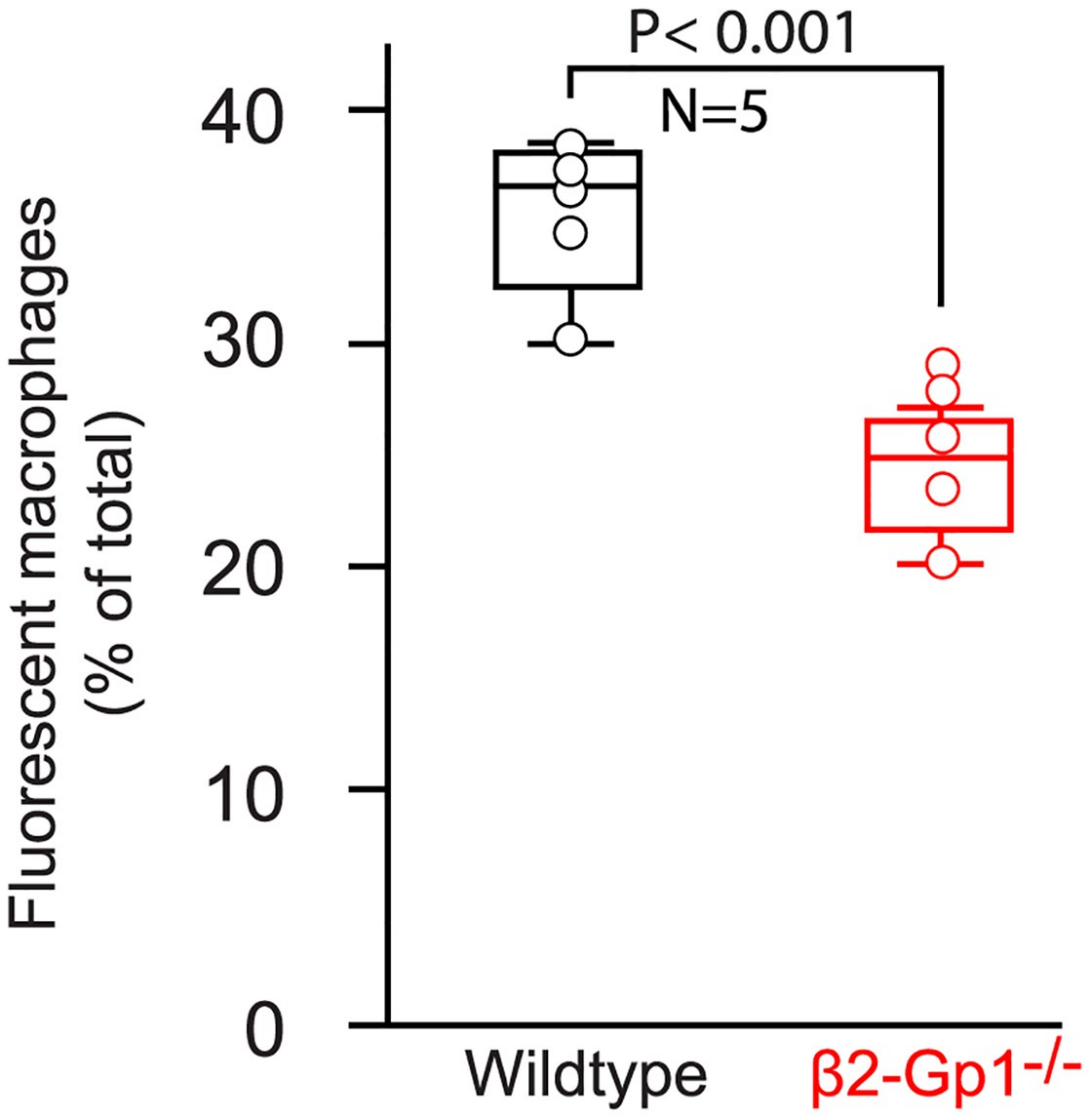
Phagocytosis of exogenous CFM in vivo. Fluorescent mitochondria, isolated from the liver of mito::mKate2 mice, were infused intravenously to recipient mice. After 30 minutes, splenic macrophages are isolated and examined for presence of intracellular fluorescent mitochondria by flowcytometry.

To assess phagocytosis of mitochondria in living animals, we intravenously infused fluorescent CFM from mito::mKate2 mice to β2-Gp1-deficient mice and wildtype controls. After 30 minutes the mice were euthanized and the macrophages from the spleen were assessed for intracellular fluorescent mitochondria by flowcytometry. As shown in Fig 6, β2-Gp1-deficient mice had significantly less mitochondria within the splenic macrophages (24.4 ± 2.72%) compared to their wildtype controls (35.6 ± 3.5, P<0.001, N = 5).

### Increased CFM and impaired phagocytosis in a patient with mutant β2-Gp1

**Fig 7:**
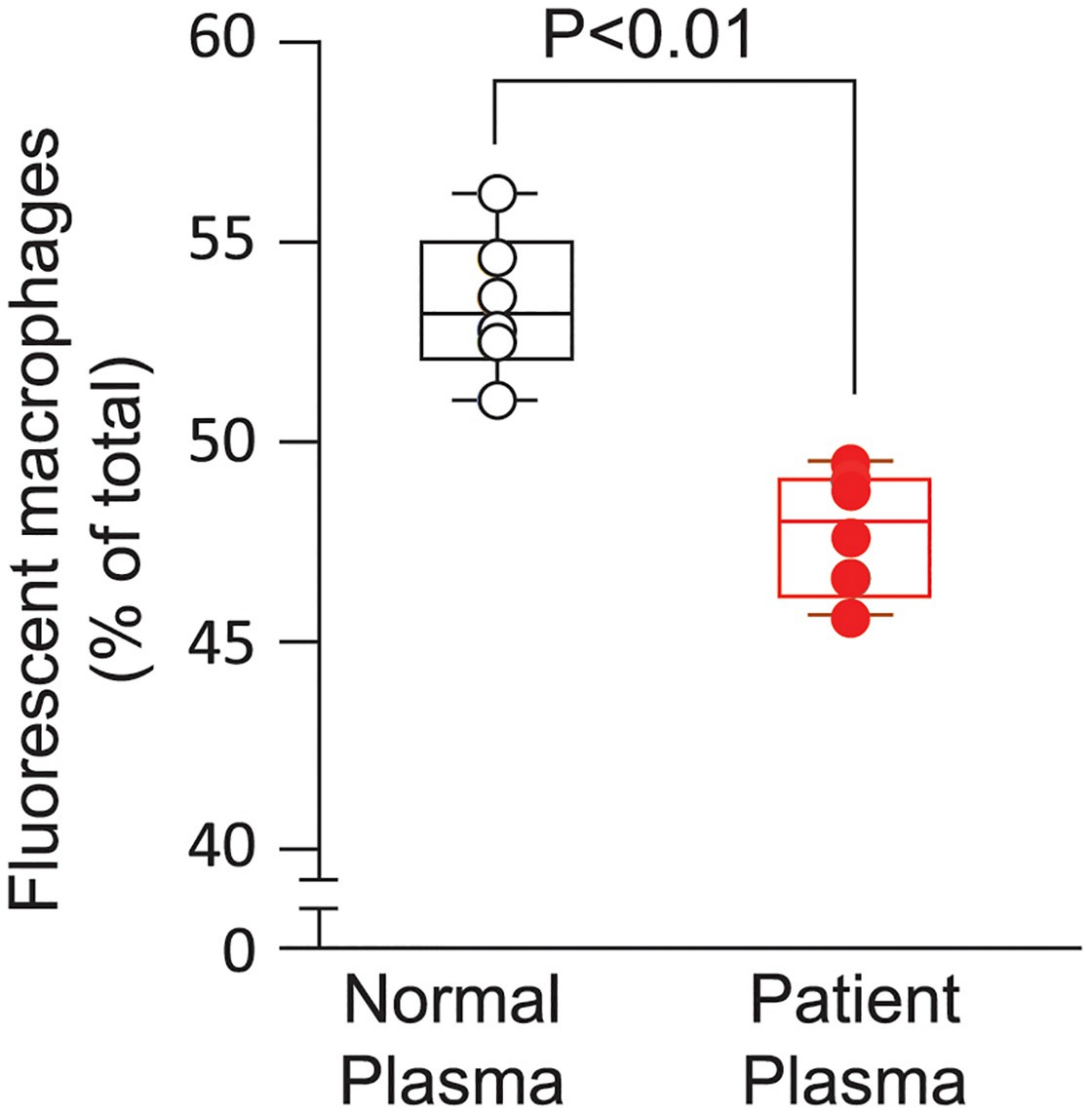
Phagocytosis of exogenous CFM. Mitochondria, isolated from HEK293, were labeled with mitoTracker green and incubated with THP-1 cell derived human macrophages in the presence of normal or patient plasma (50% v/v). After 60 minutes incubation, macrophages were washed and examined for presence of intracellular fluorescent mitochondria by flowcytometry.

A patient with nonfunctional β2-Gp1 due to compound heterozygosity to mutations in the phospholipid binding domain had increased mitochondria in the blood compared to normal controls (5.09 pg/ml versus 1.26 ± 1.35). His plasma induced less phagocytosis of CFM by macrophages (47.3 ± 1.6% versus 54.3 ± 1.3, p = 0.01) compared with plasma, from healthy controls when assessed for mitochondrial uptake by normal monocytes (Fig 7).

## Discussion

The evolutionarily conserved β2-Gp1 binds to CFM in the circulation in a cardiolipin-dependent manner and promotes its uptake by macrophages. β2-Gp1 deficient mouse has increased CFM in the plasma which increases further following endotoxin administration compared to their wildtype controls. With complete deficiency of β2-Gp1, the clearance is only moderately impaired suggesting that other cardiolipin-binding proteins such as lactadherin and Gas6 may also mediate the clearance of mitochondria, similar to multiple receptors involved in the clearance of apoptotic cells by macrophages [32]. β2-glycoprotein I binding to cardiolipin is also moderated by calcium ion [4]. Similarly, in a patient with functional deficiency of β2-Gp1, the circulating mitochondria are increased, and his plasma induced less mitochondrial uptake by macrophages compared to normal. Even though this individual [26], and others with β2-Gp1 deficiency, had unprovoked thrombosis early life, [22, 33–35], the causal relation is difficult to establish because β2-glycoprotein I deficiencies are rare. Prothrombinase activation on CFM surface could justify unprovoked thrombus formation in young patients. This patient had anticardiolipin antibodies at the time of thrombosis and he does not have any antiphospholipid antibodies currently. Thrombosis is a complex multigene phenotype and β2-Gp1 deficiency or dysfunction may be a risk factor for thrombosis in the presence of additional risk factors.

The endosymbiosis theory postulates mitochondria are derived from a symbiotic relationship of eubacteria with primitive host cells and they maintain some of ancestral bacterial characteristics, such as a double membrane, DNA with unmethylated CpG repeats, in addition to their ability to generate ATP from oxidative phosphorylation [36]. Recent investigations have shown mitochondria are present in the extracellular space as an intact organelle and as enclosed in vesicles [12–14]. Cardiac myocytes, leukocytes and platelets have been shown to release mitochondria to the extracellular space [10, 37, 38]. During tissue injury or sepsis, there is increased CFM in the circulation [16] and cardiolipin is exposed in the outer leaflet of damaged mitochondria [8]. Cardiolipin is procoagulant by providing binding sites for the assembly of coagulation proteases [39–41]. The 13 peptides synthesized in human mitochondria have formylmethionine at their N-terminus [42] similar to prokaryotic proteins reflecting the evolutionary origin of mitochondria. These peptides, if released in the circulation, activate leukocytes through formyl peptide receptor-1 invoking an inflammatory response [43]. Mitochondrial DNA is often damaged by reactive oxygen species generated in the electron transport chain. Unmethylated CpG motifs and the circular form of mitochondrial DNA resembles bacterial DNA and provokes potent type I interferon production and inflammatory cytokines including TNF-α production by damage-associated molecular pattern (DAMP) sensors that could contribute to the development of autoantibodies [16, 44, 45]–so frequently seen. Elucidation of the precise role of β2-Gp1-mediated mitochondrial clearance will help to understand the pathogenic mechanisms of antiβ2-Gp1 antibodies [46].

## Supporting information

S1 File (PDF)
