# Supplementary material for: β2-glycoprotein I promotes the clearance of circulating mitochondria
Source: PLoS One. 2024 Jan 25;19(1):e0293304. doi: 10.1371/journal.pone.0293304 (PMC10810532; doi:10.1371/journal.pone.0293304)
Supplement: S1 File — (PDF) [file pone.0293304.s001.pdf]

Supporting Information

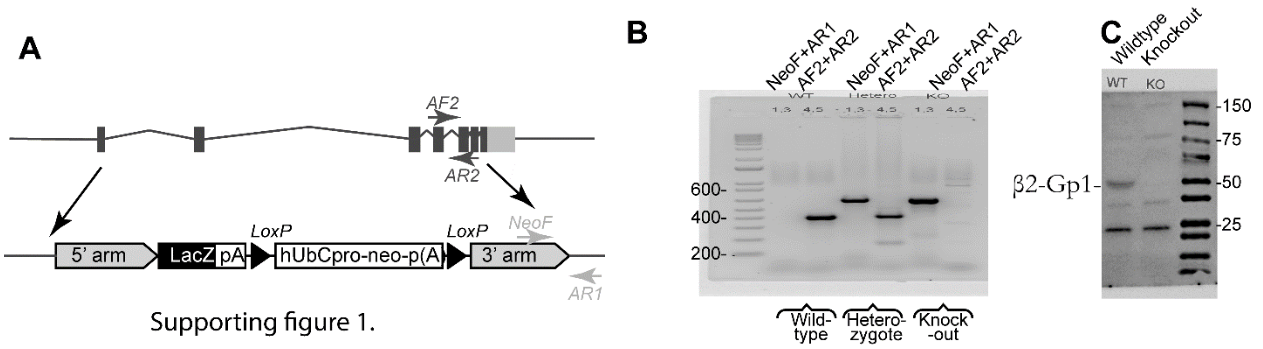

Supporting Figure 1. Characterization of β2-Gp1 knockout. Panel A. The targeting vector contains a ZEN-Ub1 cassette, consisting of LacZ-p(A) reporter and hUbCpro-Neo-p(A) selectable marker flanked by loxP sites. It replaces Exon 1-7 of β2-Gp1. Panel B. Primers AF2 and AR2 gives 400 bp in wild type only. Primers NeoF and AR1 gives 600 bp fragment in knockout only. Panel C. Immunoblot of wild-type and knockout serum with antiβ2-Gp1 antibody showing the absence of the 50 kDa β2-Gp1 in the serum of knockout animal.

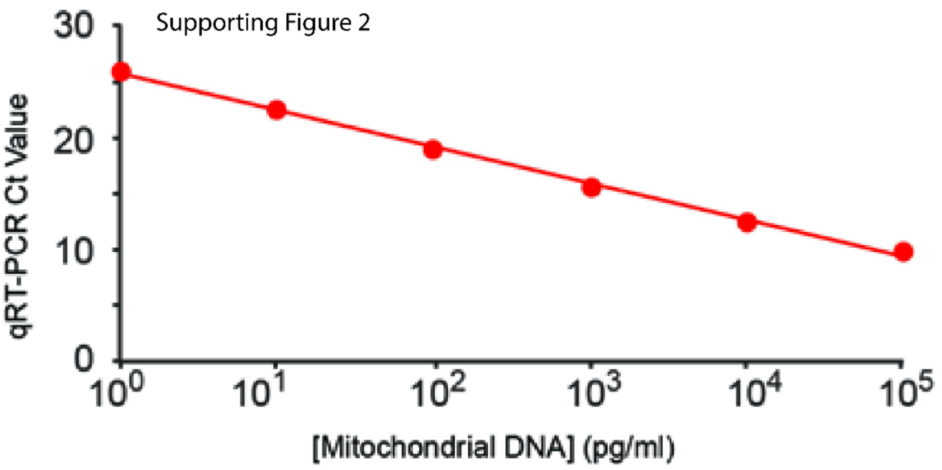

Supporting Figure 2. Quantitation of mitochondrial DNA. The curve was generated with known quantities of isolated mtDNA (mouse or human) and the corresponding Ct values obtained by qPCR. The reaction mixture contained 10 µl 2× SsoAdvanced Universal SYBR Green Supermix, 2 µl primers (10 µM) and various concentrations DNA (1pg -10 10 µg) in a final volume of 20 µl. PCR conditions were: 5 min at 95°C initial denaturation, followed by 40 cycles of 30 s of denaturation at 95°C, 15 s of primer annealing at 60°C and 10 s at 72°C of extension.

Supplementary Table I. Primers used in PCR amplifications.

primer	Sequence	Annealing temperature
AF2	5'-TGA GAC AGG GTT TCT CTG TAT AGT CCC GG	
AR2	5'-CAC TTA GAT GAG CTG GTC CCA TTC AG	65ºC
NeoF	5'-GCA GCC TCT GTT CCA CAT ACA CTT CA-3'	
AR1	5'- AAA CAG AAC AAG GGA AGG GCA GAG G-3'	65ºC
M2BALF (Balb/c mitochondria specific primer)	5'-CTG ACA TTT TGT AGA GGA AA	
M2BALR (Balb/c mitochondria specific primer)	5' AGA TAA CAG TGT ACA GGT TG-3	55ºC
MTF Mouse mitochondria-specific primers	5′-CTAGAAACCCCGAAACCAAA-3′	

MTR Mouse mitochondria-specific primers	5′-CCAGCTATCACCAAGCTCGT-3′	60ºC

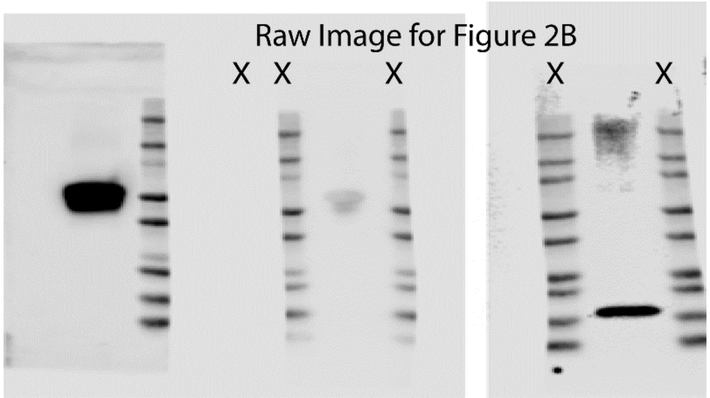

Supplementary Figure 3. Unedited Raw image of Figure 2B. The edge lanes not included in the final figure are marked “X”

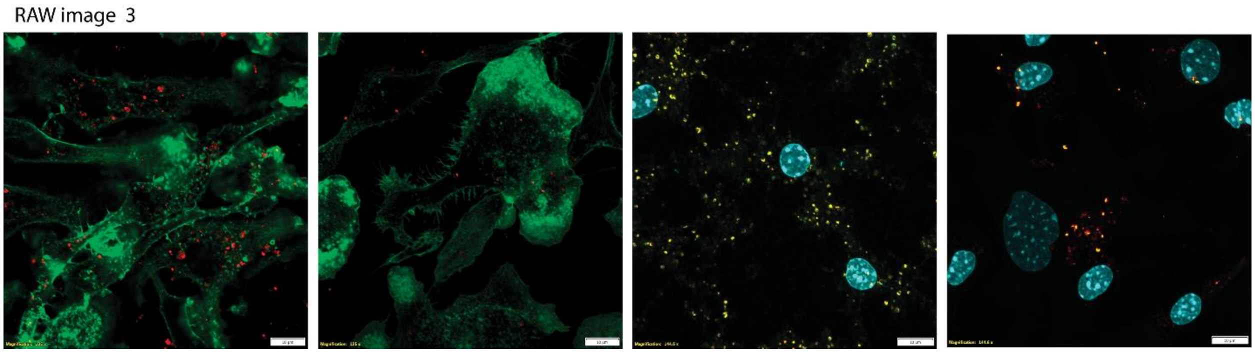

Supplementary Figure 4. Unedited Image of Figure 3.

Raw Image 5

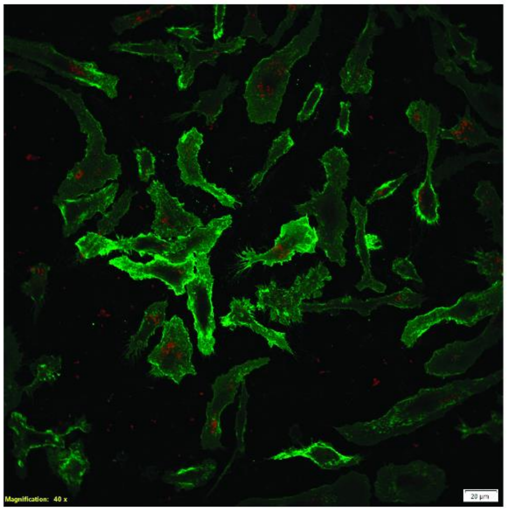

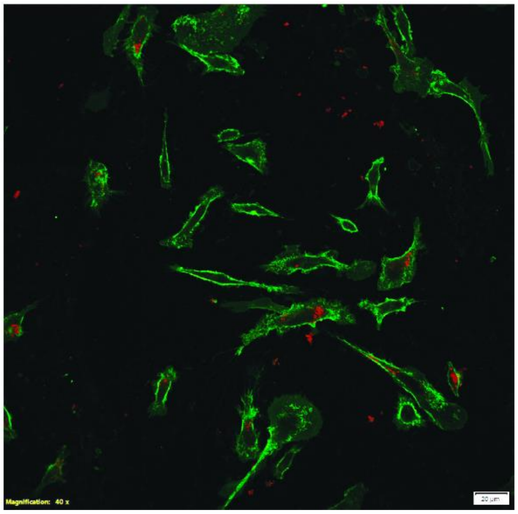

Supplementary Figure 5. Unedited image of Figure 4.
